# Light-induced reversible reorganizations in closed Type II reaction centre complexes: physiological roles and physical mechanisms

**DOI:** 10.1098/rsob.220297

**Published:** 2022-12-14

**Authors:** G. Sipka, L. Nagy, M. Magyar, P. Akhtar, J.-R. Shen, A. R. Holzwarth, P. H. Lambrev, G. Garab

**Affiliations:** ^1^ Institute of Plant Biology, Biological Research Centre, Szeged, Temesvári körút 62, 6726 Szeged, Hungary; ^2^ Institute of Medical Physics and Informatics, University of Szeged, Rerrich B. tér 1, 6720 Szeged, Hungary; ^3^ Institute of Interdisciplinary Science, and Graduate School of Natural Science and Technology, Okayama University, 700-8530 Okayama, Japan; ^4^ Institute of Botany, Chinese Academy of Sciences, 100093 Beijing, People's Republic of China; ^5^ Max-Planck-Institute for Chemical Energy Conversion, 45470 Mülheim a.d. Ruhr, Germany; ^6^ Department of Physics, Faculty of Science, University of Ostrava, 710 00 Ostrava, Czech Republic

**Keywords:** chlorophyll fluorescence, dielectric relaxation, dynamics and structural memory of proteins, Marcus theory, photosystem II, purple bacterial reaction centre

## Abstract

The purpose of this review is to outline our understanding of the nature, mechanism and physiological significance of light-induced reversible reorganizations in closed Type II reaction centre (RC) complexes. In the so-called ‘closed' state, purple bacterial RC (bRC) and photosystem II (PSII) RC complexes are incapable of generating additional stable charge separation. Yet, upon continued excitation they display well-discernible changes in their photophysical and photochemical parameters. Substantial stabilization of their charge-separated states has been thoroughly documented—uncovering light-induced reorganizations in closed RCs and revealing their physiological importance in gradually optimizing the operation of the photosynthetic machinery during the dark-to-light transition. A range of subtle light-induced conformational changes has indeed been detected experimentally in different laboratories using different bRC and PSII-containing preparations. In general, the presently available data strongly suggest similar structural dynamics of closed bRC and PSII RC complexes, and similar physical mechanisms, in which dielectric relaxation processes and structural memory effects of proteins are proposed to play important roles.

## Graphical abstract

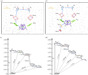

## 1. Introduction

The conversion of light energy into chemical energy by prokaryotic and eukaryotic photosynthetic organisms serves the energetic basis of virtually all life on Earth; also, fossil fuels are energy deposits of photosynthesis across millions of years; the photosynthesis of cyanobacteria, algae and vascular plants created and maintains the oxygen-rich atmosphere of our planet [1–5].

The light reactions of photosynthesis occur in photosynthetic membranes, which embed light-harvesting (LH) antenna and reaction centre (RC) complexes, mobile electron carriers, cytochrome (cyt) *b*_6_*/f* or *b/c* complex, and ATP synthase. Photosynthesis begins with the absorption of light, which predominantly occurs in the LH antenna complexes of the photosynthetic machinery. The excitation energy of the absorbed light energy is then transferred to the photochemical RCs by a series of ultrafast energy transfer processes. In the RC, primary charge separation occurs, which is followed by secondary events stabilizing the charge-separated state; these are the first steps of the photosynthetic energy conversion. These events are followed by vectorial electron transport (ET) and associated proton transfer processes—leading to the formation of an electrochemical potential gradient across the membrane, which is then used for ATP synthesis. The ET also produces reducing equivalents, which—in the form of NAD(P)H, together with ATP molecules—are consumed during the synthesis of carbohydrates from carbon dioxide. In addition, in oxygenic photosynthetic organisms, molecular oxygen is evolved from splitting of water molecules [6], which is released to the atmosphere.

The RC complexes of photosynthetic organisms are multi-subunit pigment-protein complexes, which also contain lipids, various cofactors and metals [7]. In the RCs, the initial photochemistry is catalysed by (bacterio)chlorophyll ((B)Chl) molecules with the involvement of their special pairs. The RCs can be classified, according to their electron acceptors, as Type I or iron-sulfur type and Type II or pheophytin-quinone type centres. Type I RCs comprise photosystem I (PSI) of oxygenic photosynthetic organisms and the RCs of green sulfur bacteria and heliobacteria. RCs of photosystem II (PSII) and purple and green gliding bacteria belong to Type II centres. Type I and Type II RCs differ in two further important features [8]: (i) On the donor side, Type I RCs accept electrons from water-soluble diffusible electron donors, such as plastocyanin or cyt *c*, while Type II RCs exhibit bound electron donors, such as tyrosine Z (Y_Z_) and the oxygen-evolving complex (OEC) of PSII, or the RC-bound cyt *c* subunit of some purple bacteria. (ii) On the acceptor side, in Type I RCs the mobile one-electron acceptor ferredoxin, a water-soluble protein, carries away one-by-one the reducing equivalents from the RCs. By contrast, in Type II centres the secondary, two-electron quinone acceptor (Q_B_) molecules, after their protonation (QH_2_), are released into the lipid phase of the membrane. These structural and functional differences justify the separate treatment of Type I and Type II RCs and might explain that, while numerous authors have reported marked light-induced conformational transitions in Type II RCs (see below), the conformational dynamics of PSI appears to be more restricted [9].

In this review, we summarize the current state of our knowledge about the structure and function of Type II RCs—using the atomic or near-atomic resolution models and the reaction kinetics data of the RC of the purple bacterium *Rhodobacter* (*Rb.*) *sphaeroides* (bRC) and of PSII core complex (PSII CC) of the cyanobacterium *Thermosynecochoccus* (*T.*) *vulcanus*. We also provide a brief synopsis of the primary events of closing open RCs (RC_O_) via charge separation and stabilization. These steps are followed by secondary electron and proton transport processes, some of which have been shown to be associated with reorganizations in the RC complexes—indicating in general the structural dynamics of Type II RCs. In this review, our attention will primarily be focused on light-induced alterations of closed RC (RC_C_) complexes. RC_C_ complexes are not capable of forming additional stable charge separation. Nevertheless, their repeated multiple excitations, both in bRC and PSII CC, have been demonstrated to further and substantially stabilize the charge-separated states. Considerations will be given to the underlying physical mechanisms which are believed to be responsible for the observed changes in the reaction kinetics. Special attention will be paid to the roles of stationary and transient electric fields and dielectric relaxation processes. We provide a collection of experimental observations of structural changes in bRCs and in PSII and will emphasize that the nature and mechanism of these reorganizations require further systematic investigations. We also emphasize that the observed light-induced structural changes—which lead to relatively long-lasting memory effects associated with substantial changes in various electron transfer rates—appear to be tightly linked to the general nonlinear behaviour of proteins [10–12].

## 2. Structure, reaction kinetics and structural dynamics of Type II reaction centres

Both bRC and PSII CC are composed of protein subunits and spectrally sensitive redox active cofactors. They are light-dependent enzymes catalysing, respectively, the reduction of ubiquinone (UQ) by cyt *c*^2+^ and the oxidation of water and the reduction of plastoquinone (PQ) [13].

**Figure 1.**
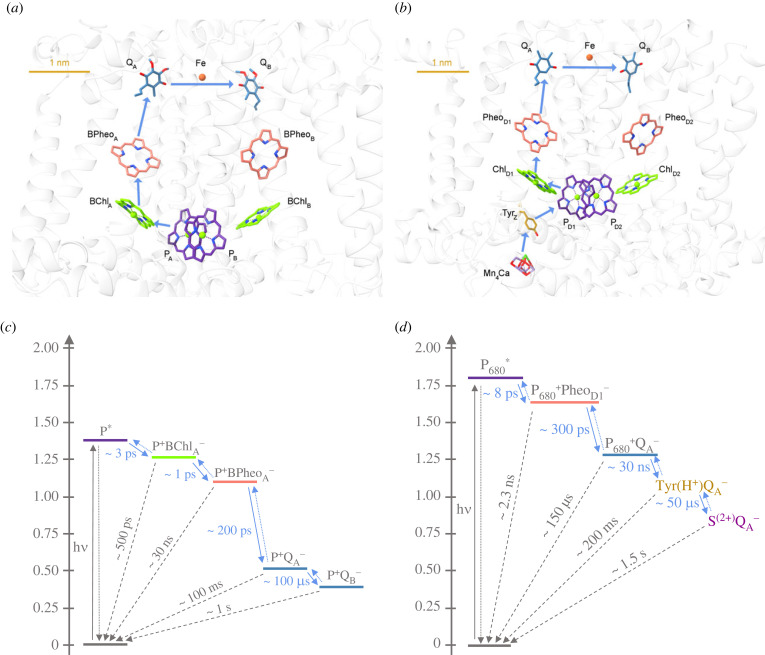
Cofactor structures (*a*,*b*) and routes of charge separation and approximate electron transfer time constants (*c*,*d*) in the RCs of model organisms *Rb. sphaeroides* (pdb:3I4D) and *T. vulcanus* (pdb:5GTH). Structure visualization with UCSF ChimeraX [17]. (Note the different scales in *a* and *b*; in *c* and *d* the states on the free energy scale are in eV; also, for simplicity, in *d* the Q_A_ to Q_B_ electron transfer is not displayed.).

The bRC complex consists of three polypeptides that are denoted L, M and H [14–16]. A single-transmembrane *α*-helix H subunit possesses a cytoplasmic domain; its elimination results in only subtle change in the basic function of the bRCs. The L and M polypeptides have five *trans*-membrane *α*-helices and accommodate all the cofactors. In *Rb. sphaeroides*, they encase four BChl-*a* molecules, two of which (denoted P_A_ and P_B_) form the P_870_ special pair, while B_A_ and B_B_ are monomers; they also encase 2 BPheo-*a* (H_A_ and H_B_) molecules, two UQs (Q_A_ and Q_B_), a single carotenoid (Crt) and a non-heme iron atom (figure 1*a*). (Indices A and B refer to A and B branches of the RC.)

PSII CC is capable of splitting water and evolving oxygen as well as reducing PQ. In *T. vulcanus*, it is composed of 17 transmembrane subunits, three peripheral proteins, thylakoid membrane (TM) lipids, Chl-*a* and *β*-carotene molecules and several other atoms/ions and molecules, including bicarbonate and the Mn_4_CaO_5_ cluster of the OEC—with a total molecular mass of 350 kDa for a monomer of the homodimeric supercomplex [18]. It contains the protein heterodimer D1/D2 (homologous to L/M of the bRC), the *α* and *β* subunits of cyt *b_559_* and the two integral antenna proteins, CP43 and CP47, which carry 14 and 17 Chl-*a* molecules, respectively. The arrangement of the basic cofactors in the D1/D2 PSII RC is similar to that in the bRC: two of the four Chl-*a* molecules, P_D1_ and P_D2_, are analogous to the special pair P_A/B_; the two ‘accessory' Chls (Chl_D1/D2_), two Pheos (Pheo_D1/D2_), a non-heme iron and two PQs (Q_A_ and Q_B_) are arranged in two pseudosymmetrical branches. In addition, the D1/D2 proteins also accommodate the redox-active tyrosine residues Y_Z_ and Y_D_, and CP43 is involved in the assembly and activity of the OEC [19,20] (figure 1*b*). In eukaryotic organisms, PSII also contains peripheral LH complexes attached to the CCs [21,22].

After the absorption of a photon by the RC or upon the arrival of an exciton from CP43/CP47 or LH1 (the inner LH antenna of PSII and bacterial RC) [23], the primary electron donor P_680_ in PSII or P_870_ in bRC, respectively, assume electronically excited states. (In the present review, P_680_ is referred to as the primary electron donor from which charge separation starts, irrespective of its molecular identity; for a more exact treatment, see Romero *et al*. [24]). The subsequent charge separations proceed asymmetrically, only along the D1 and A branch in PSII and in bRC, respectively. In the bRC, the first clearly identifiable radical pair $\left(P^{+}{\mathrm{BChl}}_{A}^{-}\right)$ is formed in about 3 ps, which is followed by an approximately 1 ps electron transfer step leading to the formation of $P^{+}{\mathrm{BPheo}}_{A}^{-}$ [25–27] (figure 1*c*). Excitations in the core antenna Chls of PSII lead to the accumulation of the P_680_^+^Pheo^−^ radical pair with an apparent lifetime of 30–60 ps [28–30]. The reaction comprises of several energy and probably electron transfers, whereas the intrinsic charge separation time is less than 10 ps [31–33] (figure 1*d*). In both bRC and PSII, the primary charge separations are stabilized via the re-oxidation of (B)Pheo^−^ by Q_A_, forming $P_{680(870)}^{+}{(\;B)\;\mathrm{PheoQ}}_{A}^{-}$ within approximately (200)300 ps.

On the acceptor side, these steps are followed by slower ET reactions between the primary and the secondary quinone acceptors Q_A_ and Q_B_. Meanwhile in PSII, the primary electron donor is re-reduced by electron donation from the nearby redox-active tyrosine, forming neutral tyrosyl radical $Y_{Z}(H^{+}{)\;Q}_{A}^{-}$, which is then reduced by the Mn_4_CaO_5_ cluster of the OEC, leading to $S_{2}{(}^{+})Q_{A}^{-}$, where *S_2_* denotes the state of the OEC after a single-turnover saturating flash (STSF) excitation. In bRC, the oxidized primary donor is re-reduced by an external electron donor cyt *c*_2_. In isolated systems, secondary ET (Q_A_ to Q_B_) is easily blocked—for example, in the presence of ET inhibitors or at cryogenic temperatures, or in media lacking secondary electron donor or acceptor molecules. In such conditions, the RCs are reset by recombination of the positive and negative charges (i.e. the oxidized electron donors can be re-reduced via electron tunnelling, directly or indirectly, via thermal activation). The observed rate constants are composed by linear combination of the rate constants of the relaxation paths. According to the semi-classical (ћ*ω*/*k_B_T* << 1) version of the Marcus theory (in which the molecular movements are characterized by harmonic oscillators), we can investigate the dependence of the direct charge recombination as a function of the free energy (see below). The time constants of the forward and backward reactions in bRC and PSII are displayed in panels (*c*) and (*d*), respectively, of figure 1. Theoretical descriptions and fine tuning the proton and electron transfer parameters such as free energy, electronic coupling or reorganization energy by protein engineering serves as the basis of a deeper understanding of the molecular principles underlying the photosynthetic reactions. In particular, it has been thoroughly demonstrated that protein relaxation processes occurring on the timescale of hundreds of picoseconds play important roles in the formation of stable charge separated state [34,35].

Both bRC and PSII CC have been reported to undergo reorganizations associated with secondary ET events. Time-resolved serial femtosecond crystallography experiments revealed structural changes in PSII CC of *T. vulcanus*—around the Q_B_/non-heme iron and the Mn_4_CaO_5_ cluster—induced by two-flash illumination at room temperature [36] and in the S-states of OEC using triple flashes [37,38]. Light-induced reorganizations around the Q_B_ pocket, associated with the secondary Q_A_^–^Q_B_ to Q_A_Q_B_^–^ electron transfer and protonation event, have earlier been proposed to occur in *Rb. sphaeroides* [39], which would suggest similar structural changes in bRC and PSII. However, the mechanisms of proton transfer associated with the reduction of Q_B_ appear to be different in PSII and bRC [40,41], and thus, details of the nature and mechanisms of the structural dynamics might also be different.

## 3. Theoretical background

The free energy of every individual photon (E=hν) absorbed by the RCs (or the exciton energy received from the antenna) is converted to Gibbs free energy (Δ*G*) according to

3.1 $$\begin{array}{c} \Delta G=\Delta H-T\Delta S \end{array},$$

where *H*, *T* and *S* are, respectively, the enthalpy, the absolute temperature and the entropy of the system at constant temperature and pressure. The used photon energy assures the enthalpy changes of the successive steps of the redox reactions of the ET chain in the RCs—this is the useful work for the photoelectric energy conversion. The yield of useful work (Δ*G*) and the thermal/conformational changes (entropy contribution, *T*Δ*S*) were measured for various photosynthetic systems; however, there is only limited availability of suitable techniques. A detailed description of coupling kinetic, thermodynamic and structural processes usually requires sophisticated experimental arsenal, and measurements in different measuring conditions (sample preparations and sample conditions). In addition, determining parameters of transient or irreversible (non-equilibrium or intermediate), spectrally ‘silent' species (no optical change connected directly to the chromophore) is usually difficult. Further difficulty is given by the temperature dependence of the thermodynamic parameters. There are clear examples showing that entropy plays an important role in some of the photosynthetic ET or charge recombination steps in different systems [42,43]. Further, part of the energy which is not used for photochemistry and will be dissipated as heat, might induce structural changes (cf. Cseh *et al*. [44], see also Arnlund *et al*. [45]).

In addition to the energetic requirement defined by equation (3.2), there exists also a kinetic limitation manifested in the reaction rate, *k*_et_*,* as defined by the Arrhenius equation

3.2 $$\begin{array}{c} k_{\mathrm{et}}\propto k_{R}e^{-(\Delta G^{{\;}^{\#}}/(k_{B}T))}, \end{array}$$

where *k_R_*, *T* and *k_B_* denote the distance-dependent electron transfer rate constant, the absolute temperature and the Boltzmann constant, respectively.

To describe the forward ET processes and the recombination events, Marcus theory is employed [46,47]. The rate of electron transfer (*k*_et_) between a donor and an acceptor molecule, according to Marcus theory is a function of the standard free energy (Δ*G*^0^) and the reorganization energy (*λ*). The reorganization energy corresponds to the energy that must be added to the initial equilibrium state to move the initial state to a geometry that corresponds to the equilibrium geometry of the final state without the occurring of the electron transfer reaction. The potential barrier that separates the starting molecules from the product, related to the harmonic model, is the activation energy (*E*_a_ or Δ*G*^#^) which can be calculated using the following equations:

3.3 $$\begin{array}{c} \Delta G^{{\;}^{\#}}=\frac{{\left(\lambda +\Delta G^{0}\right)}^{2}}{4\lambda } \end{array}$$

and

3.4 $$\begin{array}{c} k_{\mathrm{et}}\propto k_{R}e^{-(({\left(\lambda +\Delta G^{0}\right)}^{2})/(4\lambda k_{B}T))} \end{array}.$$

Considering the nuclear motion in a classical way and the charge transition quantum mechanically, we obtain the semi-classical Marcus equation [47]

3.5 $$\begin{array}{c} k_{\mathrm{et}}=\frac{2\pi }{\hbar }{\left|H_{AB}\right|}^{2}\frac{1}{\sqrt{4\pi \lambda k_{B}T}}\;e^{-(({\left(\lambda +\Delta G^{0}\right)}^{2})/(4\lambda k_{B}T))} \end{array}.$$

This formalism can be applied when all the vibrations are excited (above *k*_B_*T*). I*H_AB_*I is the electronic coupling between the initial and the final states.

The reorganization energy is the energy required to distort the conformation and position of both the reactant and the solvent molecules to allow the transition to occur. Thus, *λ* is the sum of the solvent-independent internal reorganization energy (*λ*_int_, inner sphere) and the solvent reorganization energy (*λ*_s_, outer sphere)

3.6 $$\begin{array}{c} \lambda =\lambda_{\mathrm{int}}+\lambda_{s} \end{array}.$$

Accordingly, the reorganizations arise from structural differences between the relaxed nuclear geometries of the reactant and the product and from differences between the orientation and polarization of the solvent molecules surrounding the reactant and the product [48]. These quantities depend on dielectric polarization processes, and the energy of transition state is predominantly the energy of the solvent. In particular,

3.7 $$\begin{array}{c} \lambda_{s}\;\tilde{}\frac{1}{\varepsilon_{\mathrm{opt}}}-\frac{1}{\varepsilon_{s}} \end{array},$$

where *ε*_opt_ and *ε*_s_, respectively, are the optical and static dielectric constants of the solvent [47].

It is important to note that the dielectric polarization processes may occur on different timescales and the overall dielectric relaxation associated with the ultrafast ET steps might be slow. This, in fact, is a salient feature of proteins, which, in contrast to more homogeneous media like liquids or even solids, possess widely distributed dielectric and conformational relaxation kinetics, very often distributed over many orders of magnitude in time [49]. This inherent feature of proteins is a very essential part of their function as a very special ‘solvent' e.g. for electron transfer cofactors that enable proteins to enhance and control electron transfer in a very special fashion, different from simple solvents. In general, this is because of the delay in molecular polarization with respect to a changing electric field. It is to be pointed out that Marcus theory ‘[does not consider] the effect of the dynamics of solvent dielectric relaxation on electron-transfer rates' [47]. In other terms, Marcus theory assumes—in its original form—that environmental or solvent relaxation occurs on a time scale at least congruent with the rate of the electron transfer process. However, dynamic effects might be of significance because of the temporal evolution of the polarization of the medium (here, the protein matrix of the RC complex) upon an essentially instantaneous charge separation event and very rapid ET. In proteins, the dielectric relaxation processes, shielding the charges and reducing the field strength, usually exhibit distributive kinetics and display several parallel and consecutive kinetic components [49].

## 4. Light-induced stabilization of the charge-separated state, structural memory

In isolated bRC of *Rb. sphaeroides*, long-lived light-induced charge-separated states were identified in which the rate of charge recombination was slowed down by up to three orders of magnitude compared to that after an STSF excitation. Such slowly recombining states were generated by continuous illumination or by trains of STSFs both in the presence and absence of Q_B_ [50]. These data—suggesting a conformational memory in the bRC—were fully consistent with the Kleinfeld effect. Briefly, functionally important, light-induced structural reorganizations, affecting both the stability of the $P^{+}Q_{A}^{-}$ charge-separated state and the $P^{+}Q_{A}^{-}Q_{B}$ to $P^{+}Q_{A}Q_{B}^{-}$ forward ET, were observed by Kleinfeld and co-workers [51,52]. It shows that slow structural motions provide a structural ‘memory' effect, and that the ‘light-adapted' conformation can be trapped at low temperature. This type of behaviour of bRCs has been explained in terms of a more general theory of a self-regulatory mechanism of photoactivated donor–acceptor molecular systems which possess the ability to undergo slow structural reorganizations [53]. The theoretical model predicted the gradual formation of a light-adapted conformational state from the dark-adapted conformation of the bRC. The transition can only be observed after repeated excitation of the sample, which is explained by the low percentage of RCs remaining structurally deformed after recombination, and thus the ‘memory-bearing' centres accumulate relatively slowly. This kind of mechanism has been shown to couple protein reactions to their slow structural dynamics [12]. The nonlinear behaviour of proteins is triggered very efficiently when the coupling between intrinsic fast reaction processes and slow conformational (relaxation) modes of the protein complexes is strong [54]. This situation is ideally fulfilled by internal electron transfer processes in photosynthetic RCs, creating extremely strong internal electric fields, which in turn can couple strongly to ions, redox active cofactors or charged amino acids in the protein [55,56].

**Figure 2.**
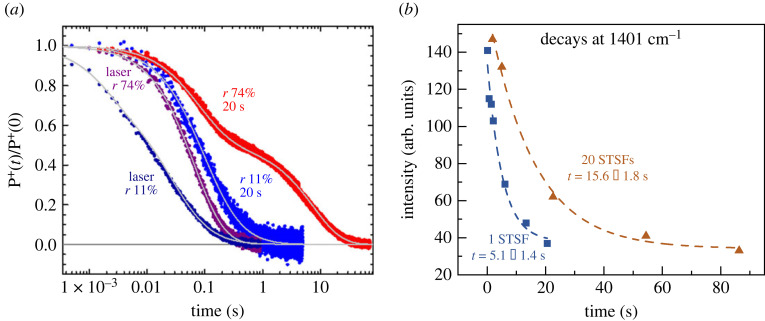
Pre-illumination dependence of the recombination rates of the charge separated states of bRC at two different hydration states (*r*) (*a*), and PSII CC (*b*), determined by absorbance kinetic transients at 422 nm (*a*) and relaxation kinetics of the FTIR signal at 1401 cm^–1^ (originating from the *S*_2_ state of the OEC) (*b*). Pre-illumination conditions: (*a*) single 7-ns (laser) flash and 20 s continuous light; (*b*) 1 STSF and a train of 20 STSFs at 10 Hz repetition rate. Sources: (*a*) is kindly provided by Dr M. Malferrari (based on Malferrari *et al*. [62]); (*b*) reproduced from Sipka *et al*. [63].

Further studies on slowly recombining states of bRC revealed that the ‘average survival time' of the charge-separated state and its dark relaxation times were correlated positively with the length and intensity of the illumination; these relaxation times exceeded by orders of magnitude the electronic recombination times in the RCs. The generation of the conformational state associated with the lengthening of the charge-stabilized state could also be achieved in the presence of inhibitor molecules blocking the electron transfer between Q_A_ and Q_B_. In this case, prolonged excitation of the sample converted the closed RC_C_ $\left(P^{+}Q_{A}^{-}\right)$ state to a different conformational state, tagged as ${\left(P^{+}Q_{A}^{-}\right)}^{*}$ [57]. This latter state may be referred to as charge-separated light-adapted state (RC_L_) or light-adapted closed state (see below). With the aid of using point mutants of *Rb. sphaeroides* and optical spectroscopy, it has been shown that the conformational states responsible for the slow charge recombination of the RC can be ascribed to light-induced changes of the local dielectric constant in the vicinity of the inactive BChl monomer [58]. It has also been shown that lipid binding to the carotenoid binding site in the R-26 carotenoid-less mutant, near the same inactive BChl, resulted in a drastic increase (by five orders of magnitude) in the lifetime of the charge-separated state [59]. The anomalous light-dependent temperature dependence of the recombination of the charge-separated state has been ascribed to changes in the hydrogen bonds in the Q_A_ microenvironment [60]. In isolated wild-type RC of *Rb. sphaeroides*, long and intense illumination induced a deceleration of the recombination of $P^{+}Q_{A}^{-}$ by more than two orders of magnitude [61]. The light-induced transitions and the stabilization of the charge separation in bRC, in addition to the duration of the photoexcitation period, also depended heavily on the hydration level of the sample, revealing the involvement of bound water molecules [62] (figure 2*a*). Most recently, Allen *et al*. [64], using RCs from *Rb. sphaeroides*, containing mutations in amino acid residues near the special pair, have demonstrated strong correlation between the slow recombination of $P^{+}Q_{A}^{-}$ after continuous illumination and light-induced proton release. Their results suggest the existence of a proton transfer pathway encompassing a network of hydrogen bonds and bound water molecules around P^+^ that stabilizes the charge-separated state.

In PSII, light-induced stabilization of the charge-separated state was observed only recently, using a train of STSFs applied on DCMU-treated PSII CC of *T. vulcanus* [63]. In this system, PSII_C_, generated by the first STSF, was converted by a series of flashes to a state named light-adapted charge-separated state (PSII_L_), which displayed a substantially decreased recombination rate between $Q_{A}^{-}$ and the *S_2_* state of the OEC than PSII_C_ (figure 2*b*). (DCMU, PSII inhibitor, *N*′-(3,4-dichlorophenyl)-*N*,*N*-dimethylurea, blocking the electron transfer between Q_A_ and Q_B_.)

Evidently, changes in the recombination rates must be the consequence of light-induced reorganizations in the RCs. Within the frameworks of Marcus theory, it is a close assumption, adopted in essence by all the above authors, that the changes originate from dielectric relaxation processes following the generation of the very strong local static and transient electric fields. It appears that the protein matrices of bRC and PSII assume the optimum dielectric environment relatively slowly and only gradually, with the assistance of additional excitations.

In broad terms, shielding the charges, and thus reducing the field strength explain the stabilization of the charge-separated states, which is a physiologically important event. It appears to be part of the light adaptation of the photosynthetic apparatus.

However, while there is no doubt about the occurrence of conformational changes and the physiological significance of the formation of RC_L_, elucidation of the nature and exact physical and molecular mechanisms require further detailed studies. In the following sections, we provide an inventory of the experimentally observed light-induced reorganizations in bRC and PSII. We also dedicate a paragraph on the role of the RCs' bound water molecules and other polarizable groups which are proposed to be involved in the structural dynamics and conformational memory of the RC matrices.

## 5. Conformational changes

There are strong indications showing that Type II RCs are highly inelastic systems, guaranteed by (i) protein structures outside the membrane-spanning region, (ii) helix dipole interactions in the transmembrane region, (iii) polar interactions between transmembrane helices, (iv) atomic packing in the transmembrane region and (v) ligand binding interactions in the donor and acceptor sites [52,65–70]. Nevertheless, numerous literature data pointed to the occurrence of light-induced reorganizations in bRCs [52,65–72] and in PSII [73,74]. However, identification of the—obviously subtle—light-induced reorganizations of RC_C_ states is not straightforward. This is most probably because there are many microscopic factors in the RCs, each of which might contribute to different extents to their overall, macroscopically observable light-dependent structural dynamics. The complexity of the problem might be implied by considering the strong molecular heterogeneity of protein matrix and the evidently large topographical variations of, for example, the polarizability of RC complexes, severely limiting the use of the dielectric constant (which is a macroscopic parameter). In the light of these arguments, it is not surprising that the observed reorganizations are apparently of different nature and origins. In addition, physical mechanisms other than dielectric relaxation, such as generation and effects of local heat packages due to dissipative events, might also be involved. Although most of the presently available data suggest the involvement of the protein matrix and bound water molecules, the possible role of lipid and pigment molecules should not be overlooked. In the following sections, we provide an overview of the occurrence of structural changes—following an approach of the primary techniques used.

### 5.1. Protein conformational changes detected by X-ray diffraction and infrared spectroscopy

Conformational rearrangements of the proteins are of special interests, because these include (i) fluctuations of intramolecular conformations, (ii) rearrangement of the dielectric medium and hydrogen bond interactions (including protonation and deprotonation of specific amino acids, and relaxation processes), (iii) translational conformational movements of components within the protein, including transition of (sub)states between dark- and light-adapted forms and (iv) functional interaction with the environment (hydrophobic, hydrophilic and salt interactions, binding water and specific reactants, receiving and sending electrons from and to the redox carriers in the environment under proper conditions) [75].

The most direct evidence for structural changes in proteins can be obtained by X-ray scattering and atomic-resolution X-ray crystallography techniques and FTIR spectroscopy. The application of the extremely brilliant source of X-ray free-electron laser (XFEL) has opened up the possibility to use pump-probe X-ray techniques to detect short-lived light-induced transient conformational states in proteins [76]. The method of detecting ‘protein quake’, according to which ‘proteins rapidly dissipate energy through quake-like structural motions' has been elaborated by Arnlund *et al*. [45]. By using time-resolved wide-angle X-ray scattering (TR-WAXS) with an XFEL, it has been shown that multiphoton excitation of *Blastochloris* (*B.*) *viridis* RC leads to ‘an ultrafast global conformational change that arises within picoseconds and precedes the propagation of heat through the protein'. In these experiments, on average, each RC encountered about 800 photons during the 500-fs near-infrared pump pulse, inducing an instantaneous heating of the cofactors by some thousands of degrees. Under these conditions, it was found that the structural deformation induced by the dissipation propagates faster through the protein than heat. These data might suggest that the propagation of protein strains due to the dissipation of unused excitation energy are of primary significance compared to local heat effects. However, it must be emphasized that especially the secondary ET processes in RCs depend heavily on the temperature, and there are constituents, such as lipids and phytol chains of (B)Chls found in large quantities in the RC complexes; these constituents might be sensitive to transient heat packages. Such a mechanism, thermo-optic effect, has been shown to be responsible for the light-induced, dark-reversible reorganization of lipid:LHCII membrane crystals [77] (LHCII, plant LH complex II). In LHCII, local heating was generated by exciton–exciton annihilation [78], in much the same way as in the XFEL TR-WAXS experiments, and the temperature of Chl-*a* molecules was measured by the broadening of the transient absorption signal in the Chl Q_y_, which was ‘calibrated' using steady-state spectroscopy between 10 and 100 K. The cooling of the Chl-*a* molecules occurred in two phases with time constants of approximately 20 and 200 ps, attributed, respectively, to spreading the heat to the protein and transferring to the medium. These data are in reasonable agreement with the simple model of local heat jump and heat conductance [44].

The first evidence for well-discernible specific reorganizations in the ‘heart' of bRC was obtained by Wöhri *et al*. [79] using time-resolved Laue diffraction; they uncovered a 1.3 Å movement of a tyrosine side chain adjacent to the special pair of *B. viridis*. It has been proposed that the observed light-induced protonation and conformational switching of Tyr L162—via neutralizing the positive charge on P^+^—contributes to the formation of the stabilized ${\left(P^{+}Q_{A}^{-}\right)}^{*}$ (RC_L_) state. More recently Dods *et al*. [80], using XFEL, observed intense light-induced structural changes occurring on a timescale of picoseconds, followed by lower amplitude protein rearrangements accompanying the ET steps to Q_A_ in the RC of *B. viridis*. The structural perturbations have been shown to first occur upon the photooxidation of the special pair of the RC, causing a ‘knock-on effect on the protein structure owing to the light-induced redistribution of charges' [80]. In other terms, these protein reorganizations were attributed to the sudden appearance of an electric field of about 10^8^ V m^−1^, a field strength that has been shown to perturb the structure of proteins [81]. To our knowledge, similar ultrafast protein motions have not been identified in PSII RC. It is interesting to note, however, that external electric fields of about 10^5^–10^6^ V m^−1^ have been shown to modulate the polarization state and charge-stabilization of PSII at low temperatures, down to 233 K [73,82].

Light-induced protein conformational changes have also been detected by $P^{+}Q_{A}^{-}/{\mathrm{PQ}}_{A}$ difference FTIR spectra of RC films of *Rb. sphaeroides*—revealing changes both in the amide I and amide II regions and thus providing evidence for light-induced response of the protein backbone of bRC [62]. As pointed out above, these reorganizations also depend on the hydration state of proteins. Similar protein conformational changes, affecting the amide I region of PSII CC of *T. vulcanus*, were shown to occur upon the PSII_C_-to-PSII_L_ transition under physiologically relevant conditions [63].

### 5.2. Pressure studies

The highly inelastic structure of the RC protein is demonstrated also by the fact that the protein strongly resists against external mechanical forces. High-pressure studies show that the RC of *Rb. sphaeroides* R-26.1 does not lose its three-dimensional structure at room temperature up to 0.6 GPa. However, a number of local reorganizations, specifically, in the binding site of the primary electron donor are found in the range of the atmospheric pressure to 0.2 GPa, as evidenced by Fourier-transform resonance Raman and electronic absorption spectra [83]. High pressure appears to rigidify the RC in a similar manner as cryogenic temperatures [84]. Comparison of the effect of high pressure on the carotenoid-containing bRC isolated from *Rb. sphaeroides* strain 2.4.1 with its carotenoid-less counterpart, isolated from strain R-26.1 indicated that the cavity created by the absence of carotenoid contributes to localized differences in protein compressibility. The stability of the electronic transitions of the primary electron donor under high hydrostatic pressure is observed, dependent on the presence of the carotenoid cofactor [85]. The temperature dependence of the heterogeneous spectral response of the special pair from the L- and M-branches was observed due to anisotropic build-up of the bRC protein structure purified from *Rb. sphaeroides* [86]. In general, these data show that the molecular architecture of bRCs allows limited but well-recognizable reorganizations of bRCs.

The relatively small and much larger reversible red shift of Chl Q_y_ and *S_0_*→*S_2_* carotene absorption bands, respectively, were observed as induced by high pressure up to 300 MPa on D1-D2-cyt *b_559_* complex at 277 K which is assigned to excitonically coupled Chl [87].

Further experiments on the high-pressure and cryogenic-temperature dependences of conformational transitions in bRC and PSII may contribute to the clarification of the nature of reorganizations accompanying the RC_C_-to-RC_L_ transitions.

### 5.3. Photoacoustic spectroscopy and thermal grating

Pressure or density variations (acoustic waves) propagating in the molecular environment can be tested directly by sensitive detectors (typically by piezoelectric microphones), using the techniques of photoacoustic spectroscopy. The change in the molecular structure which results in sudden change in the molecular volume (expansion or shrinkage) is a marker of the photoactivity and can be detected directly in a time resolved manner [71,75,88–90]. As pointed out above, this technique provided evidence for different light-induced volume changes / reorganizations in different photochemical RCs and is suitable to deliver quantitative thermodynamic parameters, such as enthalpy, entropy and volume changes, of the light-induced structural changes (reviewed by Hou [42]).

Another form of ‘spectrally silent' signatures of light-induced reorganizations in RC complexes is transient grating (TG), which can detect structural changes in systems containing molecules that are susceptible to local heat transients usually without exhibiting optically detectable response of the chromophore system. TG technique is a laser spectroscopy method based on detecting interference of two coherent light beams due to different light–matter interactions and subsequent photophysical and photochemical processes [91]. TG signal is given rise upon the induction of a spatially modulated refractive index (optical grating) which then diffracts another probe light beam. The diffraction is due to the change in the refractive index (*δn*) in the sample beam of the probe after pulsed light excitation by several processes. *δn* mainly comes from the released thermal energy (thermal grating, *δn*_th(*t*)_) and from the species grating *δn*_spe(*t*)_, the latter is the change in the absorption spectrum (population grating) and change in molecular volume (volume grating).

The species grating signal can be determined by the difference in *δn*_R(*t*)_ (reactant) and *δn*_P(*t)*_ (product), so that the observed TG signal (*I*_TG(*t*)_) is expressed as

5.1 $$\begin{array}{c} I_{\mathrm{TG}(t)}\;=\alpha {\left\{\delta n_{th(t)}\;+\delta n_{spe(t)}\right\}}^{2}=\alpha {\left\{\delta n_{th(t)}+\delta n_{P(t)}-\delta n_{R(t)}\right\}}^{2}, \end{array}$$

where *α* is an instrumental constant. The unique usefulness of the method is that not only the transient thermodynamic and kinetic parameters of the reactants and products but those of the intermediary species can be determined in a single measurement [92,93]. Traditionally, either the thermodynamic or the kinetic parameters of intermediates are determined.

Ultrafast kinetic techniques of this kind provide useful information about short-lived kinetic components. However, their exact interpretation requires assumptions about equilibrium conditions, such as the thermodynamic characteristics of intermediates or diffusion reactions [94]. Laser-induced transient photothermal and grating phenomena proved to be suitable for providing transient structural and thermodynamic information directly about bRCs [95,96]. It was demonstrated that the PBPheo → P^+^BPheo^–^ charge separation induced a sizeable structural change in the protein that relaxed much slower (28 µs) than the P^+^BPheo^–^ → PBPheo charge recombination (10 ns), observed in the absence of Q_A_. To our knowledge, the technique of TG has not been applied on PSII.

### 5.4. Variable (B)Chl fluorescence

Purple bacterial cells upon their exposure to intense rectangular excitation follow a relatively simple rise (induction) and relaxation kinetics, which are conventionally interpreted using the relation between the fluorescence yield and the fraction of closed RCs [97,98]. According to this, the fluorescence yield in open and closed bRCs exhibit the minimum (*F*_o_) and maximum (*F*_m_) levels, respectively; and the variable fluorescence (*F*_v_ = *F*_m_−*F*_o_) is ascribed to variations in the PQ/P^+^Q_A_^–^ redox state of the RC. In longer exposures, the fluorescence yield is also governed by ET steps on the donor and acceptor sides; and under certain conditions, additional quenchers, such as a carotenoid triplet, might affect the kinetics [99,100].

In a recent study, Maróti *et al*. [101] recorded simultaneously the induction and the relaxation kinetics of the fluorescence yield and the oxidation level of P—using short laser-diode probing flashes and transient absorption spectroscopy, respectively—in intact purple bacterial cells. The carefully selected experimental conditions—using *cycA* mutant of *Rb. sphaeroides*, lacking the natural electron donor cyt *c*_2_, and applying short excitation flashes to avoid charge recombination during the induction period—permitted to scrutinize the correlation between the concentration of P^+^ and the fluorescence yield. These measurements revealed that during the induction period the fluorescence rise was lagging behind the accumulation of P^+^; by contrast, the relaxation of fluorescence occurred faster than the reopening of the RCs (P^+^–P). The authors ascribed these deviations between the two kinetics to connectivity between the photosynthetic units (PSUs), i.e. to an exchange of excitation energy between PSUs, as also proposed for PSII units [102,103]. While connectivity between PSUs with adjacent LH1s [104] and its effects on *F*_v_ cannot be ruled out, the authors of this review think that deviations from the strict correlation between the fluorescence yield and the concentration of P^+^ might, at least in part, contain contributions from conformational changes. As shown in previous paragraphs, RC_O_-RC_C_-RC_L_ transitions generate reorganizations in bRCs. The magnitude and mechanism of connectivity can, in principle, be determined by monitoring the excitation energy migration pathways in intact chromatophores, e.g. by two-dimensional electronic spectroscopy, which is capable of monitoring both down-hill and up-hill excitation energy transfers [105]. (For comment on connectivity between PSIIs, see below.)

The primary source of *F*_v_ in cyanobacteria, algae and green plants is PSII [106]; minor contributions from PSI emission, peaking around 730 nm, have recently been identified [107]. The fast Chl-*a* fluorescence induction kinetics in oxygenic photosynthetic organisms is more complex than in purple bacteria. PSII displays a multiphasic, so-called O-J-I-P kinetics, with O and P corresponding to *F*_o_ and *F*_m_, respectively [108]. In the presence of DCMU, the kinetics becomes simpler, but the *F*_o_-to-*F*_m_ rise still follows a rather complex, sigmoidal rise.

**Figure 3.**
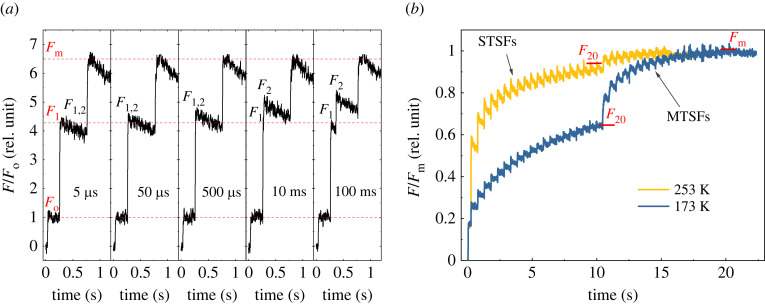
(*a*) Kinetic traces of Chl-*a* fluorescence yield upon a double-STSF excitation followed by multiple-turnover saturating flashes (MTSF) to reach the *F*_1,2_ and the *F_m_* levels, respectively, in PSII core complexes from *T. vulcanus* at room temperature in the presence of 40 µM DCMU, using different waiting times between the first and the second STSFs. (*b*) Chl-*a* fluorescence induction kinetics of DCMU-treated PSII CCs of *T. vulcanus* at 253 and 173 K.

According to the ‘Q_A_ model' of Duysens & Sweers [97], similar to purple bacteria, *F*_v_ of PSII reflects solely the reduction of Q_A_: ‘in order to reach *F*_m_, it is necessary, and sufficient, to have Q_A_ completely reduced in all the active PSII centres' [109]. Correspondingly, the *F*_v_/*F*_m_ parameter in dark-adapted sample is equated with the maximum quantum efficiency of PSII photochemistry [110–112], and the sigmoidal rise is attributed to the connectivity of PSII units [102,103]. However, it has been shown that this model is not free from controversies. The most notable discrepancy was that—in contrast to the expectations—*F*_m_ level could not be reached either during the so-called photochemical (O–J) phase [113] or by one STSF excitation which reduced all Q_A_ in the presence of DCMU, and a train of additional STSFs were required to reach *F*_m_ [114]. Magyar *et al*. [115] discovered that the efficiency of the additional flashes, gradually raising the fluorescence levels in DCMU-treated samples, strictly depended on sufficiently long Δ*τ* waiting times between flashes (figure 3*a*). The Δ*τ_1/2_* values were found to be in the order of several hundred microseconds, thus comparable with Q_A_–Q_B_ ET times. This finding qualitatively explains why the J level, at around 2 ms, remains significantly smaller than *F*_m_, despite that during 2 ms each RC may receive dozens or hundreds of excitations [116–118]. (Note that during the O-J phase, the fluorescence level is evidently lagging behind the reduction of Q_A_.) Obviously, the *F*_1_/*F*_m_ levels—which, in DCMU-treated plant TMs and cyanobacterial PSII CC, at physiological temperatures, are typically as low as approximately 0.6 and approximately 0.35, respectively [115]—cannot and should not be equated with the quantum efficiency of PSII (*F*_1_, fluorescence level elicited by the first STSF). More recently, Laisk & Oja [119] ascertained that illumination of PSII_C_ generates sizeable fluorescence rise in intact sunflower leaves. There are further clear examples—mutant cyanobacteria and the green alga *Chlorella ohadii*—showing that efficient functioning of PSII is not accompanied by sizeable *F*_v_/*F*_m_ [120,121].

The other cornerstone of the Q_A_ model is the connectivity of PSII centres, which, within the framework of Q_A_ model, is required to explain that the rise is sigmoidal, rather than exponential [103]. However, as it was pointed out in [122], a sigmoidal rise might also originate from two kinetically overlapping exponentials. In fact, the experiments with trains of STSFs indicate that several consecutive light-induced events (exponentials) follow each other [114,115]. It has also been reported that the sigmoidicity of fluorescence rise depends on the length rather than on the integral intensity of the excitation pulses: the rise is sigmoidal with a 50 µs long flash but exponential with a 2 µs flash of the same integral intensity [123]. These data are consistent with the role of waiting times between consecutive excitations. Further, DCMU-treated isolated dimeric and monomeric PSII CCs of *T. vulcanus* exhibited sigmoidal *F*_v_—showing that sigmoidal rise of *F*_v_ does not require PSII connectivity [63,115].

To obtain information on the nature of waiting times associated with the *F*_1_-*F*_2_-*F*_3_-…-*F*_m_ fluorescence increments and the underlying physical mechanisms systematic STSF-induced investigations were carried out on DCMU-treated PSII CC of *T. vulcanus*, and on intact and TRIS-washed isolated plant TMs and intact cyanobacterial cells [115]. Temperature dependence measurements revealed gradually diminishing *F*_1_ levels combined with increasing numbers of STSFs to reach *F*_m_ (figure 3*b*), and longer dark relaxation times of the *F*_m_ level with temperature decrease, and that the *F*_1_ levels remained stable below about 250 K [115]. Remarkably, *F*_m_ relaxed even at 80 K [63]. The activation energy values of the *F*_m_ relaxation (to or towards *F*_1_)—determined in the temperature ranges with no *F*_1_ relaxation—were 11.3 and 13.8 kJ mol^−1^ for PSII CC and TMs, respectively [115]. These values compare well with those obtained for two mathematically deconvoluted components of the fast fluorescence rise (13 and 16 kJ mol^−1^); the third kinetic component was essentially activation-less (approx. 2 kJ mol^−1^) [118]. It is noteworthy that the structural changes in *Rb. sphaeroides* RCs were found to decrease the free energy gap between P^+^ and Q_A_^–^ by about 12 kJ mol^−1^ [68,79]. Further, the activation energy values in PSII are also commensurate with the activation energy value (11.3 ± 0.9 kJ mol^−1^) calculated from Arrhenius kinetic analysis of the light-induced fluorescence quenching of lamellar aggregates of LHCII [124] that also undergo light-induced, dark-reversible reorganizations [77,125]. In general, the above data on PSII provided clear evidence on the involvement of conformational changes in *F*_v_.

Regarding the origin of the waiting time, it has been clarified that: (i) Δ*τ* waiting time is required between each consecutive STSFs; (ii) however, dark relaxation of the sample does not lead to the observed fluorescence increment, which thus cannot be ascribed to the release of an unknown quencher; (iii) Δ*τ_1/2_* (approx. 1–2 ms) in PSII CC was found to be almost two orders of magnitude longer than the recovery time of P_680_^+^, and (iv) the STSFs required to induce the increments produced only rapidly (*t*_1/2_ ∼ 2 ns) recombining P_680_^+^Pheo^–^ radical pairs [126]. These results clarified that the light reactions after the formation of a stable charge separation differ in their basic characters from that induced by the first STSF—and led to the formation of PSII_L_, the light-adapted closed state of the RC complex of PSII (see also §3). Note that PSII_L_ in its features resembles bRC_L_. It would be interesting to investigate whether the formation of bRC_L_ depends on waiting times between excitations, in a manner similar to PSII.

To characterize the effects of PSII_O_-PSII_C_-PSII_L_ transitions on the distribution of excitation energy in PSII CC (cf. Shibata *et al*. [127]), fluorescence emission spectroscopy experiments were performed at 80 K [63]. These studies revealed that (i) at this temperature, the *F*_o_ and *F*_1_ integrated intensities differed not more than 10–15%, i.e. the largest part of *F*_v_ originated from the PSII_C_-PSII_L_ transition (which required about 1000 STSFs); and (ii) the spectral distribution was substantially altered in PSII_L_ compared to PSII_O_ and PSII_C_. These experiments revealed three additional features: (iii) resemblance of the spectral distribution of PSII_O_ recorded at 90 K to that of PSII_L_ at 80 K, suggesting that the photothermal/thermo-optic effects—most probably arising from charge recombination—play a significant role (see 4.1); (iv) after reaching *F*_m_ at low temperature (e.g. at 190 K), the structural changes can be annealed by raising the temperature to 230–250 K; (v) in analogy with the Kleinfeld effect and structural memory in bRCs (see §3), the magnitude of the spectral changes of PSII_C_-PSII_L_ transition at 80 K depended heavily on the pre-illumination history of PSII CC at around 230 K. It is proposed that—because of the rigidity of the protein matrix, especially at lower and cryogenic temperatures—the optimum dielectric environment/polarization state of all key components require several perturbations by transient electric fields possibly combined with thermal assistance; these can be facilitated by the ‘structural memory' of the system.

Time-resolved fluorescence spectroscopic measurements recorded at 273 K on PSII CC uncovered further details about the origin of the PSII_O_-PSII_C_-PSII_L_ transitions [63]. It was shown that the decay kinetics in the *F*_1_ state was closer to that in *F*_o_ than in *F*_m_ state, revealing that the *F*_1_–*F*_m_ increment was associated with changes in the excited-state lifetime of Chl-*a*. These data confirm that the *F*_1_–*F*_m_ fluorescence yield increments are caused by altered Chl-*a* de-excitation pathways, most likely involving non-radiative recombination of transiently generated radical pairs, as proposed by Szczepaniak *et al*. [34].

Regarding the underlying physical mechanism, the presently available data on PSII are consistent with the most widely accepted view about the role of local electric fields and dielectric relaxation processes in the conformational transitions in bRCs. Dielectric relaxation processes with a broad range of lifetimes, and different dominance at different temperature intervals have been shown to occur in hydrated proteins [49]. Electric fields have been shown to affect the functioning of enzymes [128], including PSII [73,119,129,130]. These effects might be combined with thermal effects either by direct dissipation of the absorbed light energy or due to recombination of the charge separated states. Recent data—revealing the shortening of the Δ*τ* waiting times in isolated PSII CC upon the addition of TM lipids, reaching the same value as in intact TMs—point to the (i) physiology of the rate-limiting steps in the structural dynamics of PSII, and (ii) to the direct role of lipid molecules to transduce protein motions and/or participate in heat conductance processes [131]. As indicated in §3, lipids are capable of playing substantial roles in the structural dynamics of bRCs [59]. In this context, it is also interesting to note that lipids have been shown to facilitate the charge stabilization and the Q_A_-to-Q_B_ electron transfer [132,133], and that the half-time of this electron transfer step [134], at least in PSII, is commensurate with the waiting time between STSFs. It may thus be hypothesized that the dielectric relaxation in the RC complex, via shielding the charges around $Q_{A}^{-}$ and reducing the field strength, assists the Q_A_-to-Q_B_ electron transfer.

Given the fact that Chl-*a* fluorescence induction and relaxation kinetics carry information about a wide range of phenomena and mechanisms in the photosynthetic machinery—including ET kinetics, the regulation of state transitions via sensing the redox state of the PQ pool, the presence of ET inhibitors, photoinhibition and repair mechanisms, responses to biotic and abiotic stresses, and about the utilization or dissipation of the absorbed excitation energy and monitoring of photosynthetic functions in oceans [135–142]—the structural dynamics of PSII should be cautiously taken into consideration.

## 6. Physical mechanisms

The generation of the continuous-light induced long-lived P^+^Q_A_^–^ state in bRCs and the stabilization of the charge-separated state in PSII, induced by continued excitation of PSII_C_, might be complex and might involve multiple physical and molecular mechanisms. Distinct mechanisms might apply for the light-adaptation processes in bRC and PSII, as well as for the observed conformational changes and structural memories in bRC and PSII. This assumption can be justified by the substantial structural and functional differences between the two systems. While the acceptor sides are very similar (figure 1*a*,*b*), the donor side of PSII contains redox-active tyrosine residues, Y_Z_ and Y_D_, and the OEC, which ‘replace' the cyt *c*_2_ electron donor in bRC. Further, the *F*_v_ transients, involved in the dark-to-light transitions, exhibit strikingly different features, with significantly higher complexity in PSII compared to bRC (see 4.4). Nevertheless, there might be common structural and functional motifs and common fundamental physical mechanisms underlying the phenomena observed in Type II RCs.

As pointed out above, the long-lived P^+^Q_A_^–^ state in bRCs has been shown to be correlated with proton-release capability of a hydrogen-bond network, formed by amino acid residues and bound water molecules near P [64]. Other non-conflicting observations strongly suggest the crucial roles of strong, local electric fields and dielectric relaxation processes in the same process (see §3). This latter mechanism is based on the presence of polarizable groups in the RC matrices, which is evidently warranted by the set of protonatable residues and bound water molecules near P.

**Figure 4.**
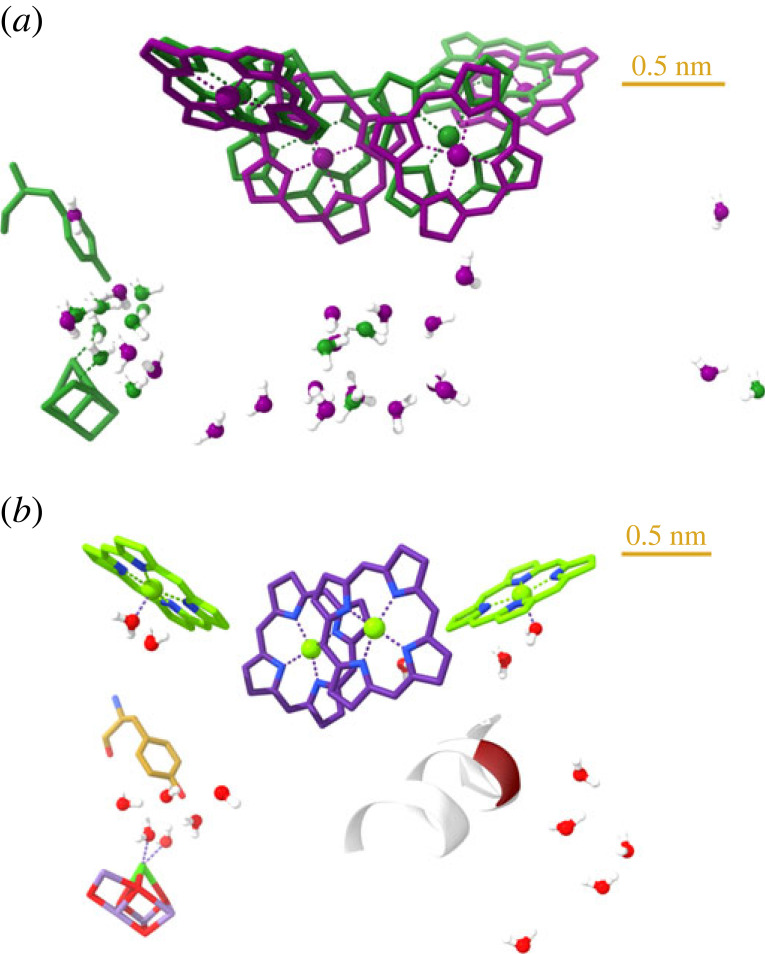
Cofactor structures and bound water molecules at the donor sides of the RCs from the model organisms *Rb. sphaeroides* (pdb:3I4D) and *T. vulcanus* (pdb:5GTH) (three-dimensional view in the electronic supplementary material). (*a*) Superimposed bRC (purple) and PSII RC (green) cofactors and bound water molecules within 3 Å distances from the amino acid residues which were proposed to be involved in a proton release pathway [64]; and PSII bound water molecules within 3 Å from the same water molecules in the bRC. (*b*) *Thermosynecochoccus vulcanus* Chl-*a* chromophores, OEC, D1-Tyr161 and the backbone of the amino acid residues, between 179 and 186, of the luminal D2 loop; the Phe181 position is highlighted. *F*_v_ mutants were generated in this section from *Synechocystis* sp. PCC 6803 [121]; each of the depicted bound water molecules is within 3 Å distance from an amino acid residue, a chromophore or the Y_Z_.

Key role of dielectric relaxation processes was also proposed in PSII to account for the rate-limiting steps and the gradual formation of the light-adapted charge-separated state [63,115]. As shown in figure 4*a*, the donor side of PSII contains sufficiently large number of bound water molecules which are found in positions near the ones located in the bRC. These water molecules, in principle, can warrant high polarizability of the RC matrix of PSII. Furthermore, it has been well established that the electron transfer reactions of the redox-active tyrosines Y_Z_ and Y_D_ are coordinated with proton transfer, suggesting the existence of a hydrogen-bonding network in their vicinities [143–145]. These data strongly suggest that the stabilization of the charge-separated state in PSII, i.e. the gradual formation of PSII_L_, might occur with a mechanism similar to that in the bRC.

On the other hand, it has been shown that the PSII_C_-to-PSII_L_ transition is closely associated with the *F*_1_-to-*F*_m_ increment of *F*_v_ [63]. In this context, it is interesting to identify two factors, associated with relatively large assemblies at the donor side of PSII, which modulate *F*_v_. (i) The period-four oscillation of the fluorescence yield indicates strong dependence of the fluorescence yield on the S-states of the OEC, both in the presence and absence of Q_A_^–^ [146]. (ii) The magnitude of *F*_v_ has been shown to be suppressed and/or substantially modulated in different cyanobacterial D2-loop segment mutants in each of which Phe181 was replaced by Trp; in these mutants, the functional activity of PSII and OEC were hardly affected while the magnitude of *F*_v_ could be as low as 10–15% of the wild-type value. The exact mechanisms of these modulations of *F*_v_ remain to be identified. It can be hypothesized that in both cases proton-transfer pathways and/or bound-water positions are affected. In the case of period-four oscillation, the S-state dependent release of protons appears to offer a relatively simple explanation [146]. For the F181W mutants, the situation might be more complex and appears to involve variations in the interactions of different amino acid residues with the RC Chl molecules and the redox-active Tyr_D_, which also affect the recombination and de-excitation pathways [121,147]. To indicate the possible roles of bound waters in these processes, figure 4*b* shows their presence in the vicinity of OEC and the mutated section of the D2 lumenal loop.

As to the physical mechanisms beyond these modulations of the Chl-*a* fluorescence and the formation of PSII_L_, additional factors, such as the required multiple excitations, the Δ*τ* waiting times, the sharp temperature dependences, additional structural factors (such as lipid molecules) and memory effects must be taken into consideration—suggesting that the reorganizations occur in relatively large moieties at the donor side of PSII. As pointed out above, also relatively large subdomains of the donor side of bRC are responsible for the stabilization of the charge-separated state in bRCs, rather than a single well-identifiable molecule.

It is proposed that—albeit the actual players participating in the gradual formation of the light adapted states in bRCs and PSII are different and may also vary between different families of photosynthetic organisms—dielectric relaxation processes represent a common physical mechanism that accounts for the fundamental phenomena. This mechanism operates with the macroscopic parameter, relative permittivity of the sample (or of its subdomain); thus, it cannot consider individual molecules or residues. However, it easily accounts for the stabilization of the charge-separated state: the dielectric relaxation processes—shielding the charges and polarizing the RC matrix—decrease the field strength and optimize the dielectric medium of the RC in its light-adapted functional state. Dielectric spectroscopy measurements and Stark-spectroscopy experiments [148] on RCs trapped at low temperature (less than or equal to 77 K) in different states would be of high interest; the characterization of such trapped states with other techniques, including fluorescence lifetime measurements and two-dimensional electronic spectroscopy techniques [149,150] would most certainly advance significantly our understanding on the consequences of the formation of light-adapted states. It might be valuable for example to exploit the capability of two-dimensional electronic spectroscopy of mapping electronic-vibrational coherences in the RC [151–154] to identify molecular adjustments in the RC associated with dielectric relaxation.

It is also interesting to point out that the dielectric behaviour of the RC protein matrices as electret material readily explains their structural memories. Electrets are dielectric materials which retain quasi-permanent electric charges or dipole polarizations. It has indeed been shown that TMs ‘remember' the combined effects of external and light-induced internal electrical fields and exhibit characteristic features of electrets [73]. Recent technical developments of laser spectroscopy make possible the use of high-intensity rectified THz pulses [155], which, in principle, offers the possibility of modulating the separation and recombination of charges in Type II RCs.

## 7. Summary and concluding remarks

In this review, we made efforts to compile literature data on the structural dynamics of closed Type II RCs—with special attention to their structural changes associated with the light-adaptation of the photosynthetic machinery. Light induced stabilization of the charge-separated state of bRCs has been thoroughly documented in the first two decades of our century. Recently, similar light-induced charge stabilization has been demonstrated in PSII CC. Regarding the nature of the conformational changes that are responsible for the gradual formation of the light-adapted states, our knowledge is more advanced for bRCs, to a large extent because of the use of recently developed advanced spectroscopic and X-ray techniques.

Concerning the physical mechanisms, the presently available data strongly suggest the central role of dielectric relaxation processes that are associated with light-induced local stationary and transient electric fields. Also, all observations are consistent with the theoretical models on the structural memory of proteins, which appears to be particularly strong for bacterial and PSII RC matrices. Thus, photosynthetic RCs are an ideal playground for studying the memory effects in proteins. The potentially high physiological/agricultural and ecological/environmental significance of the structural dynamics of PSII will most certainly motivate the research communities to open new vistas in understanding the light-induced structural and functional reorganizations of PSII, the engine of life.

## Data Availability

The data are provided in the electronic supplementary material [156].
